# Array of Horns Fed by a Transverse Slotted Groove Gap Waveguide at 28 GHz

**DOI:** 10.3390/s20185311

**Published:** 2020-09-17

**Authors:** Malcolm Ng Mou Kehn, Chih-Kai Hsieh, Eva Rajo-Iglesias

**Affiliations:** 1 Department of Electrical Engineering, National Chiao Tung University, As Well As the “Center for mmWave Smart Radar Systems and Technologies” at the Same University Under the Featured Areas Research Center Program within the Framework of the Higher Education Sprout Project by the Ministry of Education (MOE), Hsinchu 30010, Taiwan; malcolm@mail.nctu.edu.tw (M.N.M.K.); davatar320@gmail.com (C.-K.H.); 2 Department of Signal Theory and Communications, University Carlos III of Madrid, 28911 Leganés, Spain

**Keywords:** groove gap waveguide, holey EBG, millimeter wave, transverse slotted waveguide, 5G antenna

## Abstract

An array of low profile horns fed by transverse slots on a groove gap waveguide (GGWG) is presented. The GGWG is implemented with glide symmetrical holes and the design frequency is 28 GHz. The low profile horns are integrated in the same waveguide wall as the slots. The designed antenna is a linear array of these horns but the solution can be easily extended to a planar array. Experimental results support this work. The designed antenna is a good candidate for applications related to 5G technologies where medium to high gains as well as high efficiencies are required and reasonable manufacturing costs are demanded.

## 1. Introduction

Due to the low-loss properties that they exhibit, antennas based on classical waveguide technology are suitable for the millimeter-wave frequency bands. On the contrary, although printed technology allows for relatively cheap designs, the losses get too severe at high frequencies. The selection of the type of technology depends on the application and acceptable cost for a required level of performance. In new 5G networks, both technologies are feasible, as users will always require low-cost options; however, in base stations or other access points, it is possible to use a more expensive technology. Typically, waveguide technology is used to design antennas with medium to high directivity by designing arrays that usually are made with slots.

Waveguide slot arrays have many common applications, such as automotive, radar, and base station antennas [1]. Typically, longitudinal slot arrays are used, where the designs are based on locating the slots with an inter-element space of 0.5 $\lambda_{g}$. It is a popular choice due to its compactness, simplicity of the feed network, and low production cost. Transverse slot array in waveguides also constitutes a classic design [2,3], but its use has been more limited because applications requiring broadside radiation entail a spacing of a guided wavelength between two adjacent slots. As the guided wavelength is longer than the wavelength in free space, grating lobes will inevitably be generated without intervention; therefore, controlling the grating lobes is among the main challenges in the design of waveguide transverse slot arrays [4,5].

Amidst the increasing importance of millimeter wave technologies, the gap waveguide [6,7] has emerged as a novel and viable candidate for many applications within those frequency regimes. It combines the advantages of having a low loss, small size, light weight, ease of manufacturing, and low cost, thus making it suitable for antenna designs at such high frequencies [8,9]. For these reasons, arrays of transverse slots on the walls of gap waveguides are considered to be attractive solutions for high frequency array antennas in the millimeter-wave band. Many examples of arrays based on the use of corporate feed networks and multilayer strategies have been presented in the literature in recent years [10,11].

In this paper we propose the use of a transverse slot array as a way of feeding a horn array as the one proposed in [9]; however, the feed network in this paper is simplified as compared to the work in [9] where a corporate feed network made in an inverted microstrip gap waveguide was used. At the same time, this new feed network has less losses than the one in [9] as no dielectric is used and also because the groove gap waveguide (GGWG) has very low losses. Moreover, the groove gap waveguide in this paper was implemented by using glide symmetric holes instead of pins to make the technology even more cost effective. This version of the GGWG was first presented in [12] after the complete analysis of the holey structure in [13]. Some designs of millimeter wave devices using this periodic structure can be found in the literature [14,15]. The initial work related to the proposed design was presented in [16]. There is also the potential for extending this idea to planar arrays [17,18] in the future.

The paper is divided into the following sections. Mathematical modeling of a linear array of transverse waveguide slots using an equivalent network theory is presented in the next section. This is followed by the design of the transverse slot array in groove gap waveguide implemented with glide symmetric holes. In Section 4, the individual horn fed by a slot design is presented and its performance in an array was evaluated. Finally, Section 5 presents experimental results of a manufactured prototype of the design.

## 2. Theoretical Modeling

**Figure 1:**
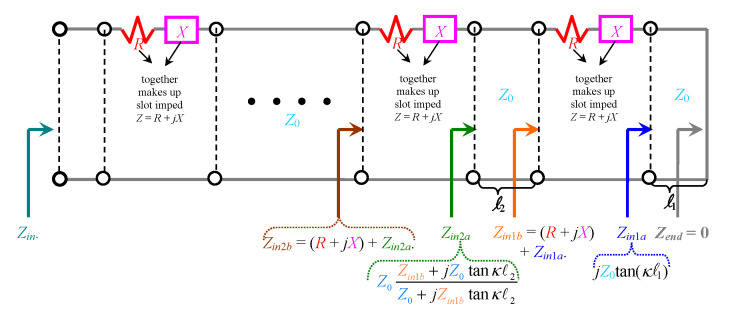
Transmission line model of a linear array of slots. Each *Z* = *R* + jX is the impedance of a slot on broad wall of a rectangular waveguide, according to [19], Part I and [20], Part II.

The equivalent impedance of slots on the broad wall of rectangular waveguides, represented as *Z* = *R* + jX, has been developed by two seminal papers [19], Part I and [20], Part II. For a linear array of such slots, the equivalent transmission line (TL) model is conveyed in Figure 1, in which $Z_{0}$ is the characteristic impedance of the TL, being in the present case, the TE-modal wave impedance of the rectangular waveguide, well-known as ωμ/β, where μ is the permeability of the material filling the guide and β is the propagation constant of the TE mode, which for the dominant $TE_{10}$ mode, is given as:

(1) $$\beta =\sqrt{\kappa^{2}-{\left(\pi /a\right)}^{2}}$$

where κ is the wavenumber (of the material filling the guide; typically vacuum) and a is the width of the waveguide. The right end of the TL is a shorted termination with load impedance $Z_{end}$ = 0 (as indicated), representative of the waveguide wall. The various lengths of TL sections separating adjacent slots are denoted by $l_{n}$ in the diagram, where *n* is just an integer index.

By basic TL theory, the input impedances at the right and left terminals of each slot can be determined, the mathematical expressions of which are given in Figure 1. For instance, $Z_{in2a}$ and $Z_{in2b}$ are those said impedances for the second slot from the rightmost one (nearest the shorted wall). In this way, the impedance looking towards all accumulated slots can be iteratively referred to the input port across the leftmost terminals, as denoted by $Z_{in}$ in the figure. This subsequently allows the reflection coefficient $\Gamma_{in}$ at that input end to be obtained as:

(2) $$\Gamma_{in}=\frac{Z_{in}-Z_{0}}{Z_{in}+Z_{0}}$$

For our case of transverse waveguide slot array, we designed the following parameters, expressed as symbols according to the notations of [19], Part I and [20], Part II:

- slot length a’ = 7.1 mm,
- slot width b’ = 2 mm,
- wall (slot) thickness t = 1 mm,
- $l_{1}$ = 7.1 mm,
- $l_{2}$ = $l_{3}$ = $l_{4}$ = $l_{5}$ = 14.2 mm, being the separation distances between adjacent slots; since there are five slots in total considered in this work, there are four such separation distances (note that $l_{3}$ through $l_{5}$ are not drawn in TL-model of Figure 1),
- $l_{6}$ = 14.2 mm = distance from left-most slot to the input port (also not drawn in Figure 1),
- Also, either of the following two parameters may apply (depending on whether the “rotated series slot” or “displaced series slot” formulas are used, either set of which yields exactly identical results. Either: displacement d = 0 (centered slot) Or rotation angle θ = $90^{\circ }$ (transverse slot)).

**Figure 2:**
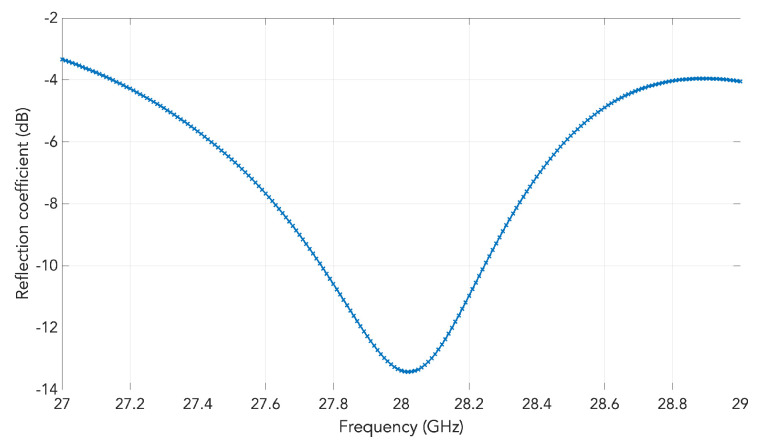
Reflection coefficient versus frequency computed by the transmission line (TL) model of Figure 1, according to [19], Part I and [20], Part II.

For the designated 28 GHz operation frequency, the computed result of the aforementioned reflection coefficient $\Gamma_{in}$ of (2) plotted against frequency for our designed case is presented in Figure 2.

## 3. Design of the Transverse Slot Array

Initially, the design of a conventional waveguide with an array of transverse slots is presented. The spacing between two adjacent slots is set to be the guided wavelength ($\lambda_{g}$). The selected frequency for the design is 28 GHz. The cross section of the 90 mm long waveguide measures 4.3 mm × 8.6 mm, which corresponds to that of a standard WR-34 waveguide, while the size of each slot after optimization is is 7.1 mm × 2 mm. A total of five slots are considered for this example.

In high-frequency applications entailing miniaturized designs, rectangular waveguides are often constructed by connecting two separate metallic pieces together, as opposed to the difficult task of drilling small rectangular tunnels through single pieces of solid cylinders. In practice, however, there are bound to be errors in the production. A common one is the occurrence of crevices due to the imperfect sealing of the separate pieces, which could subsequently cause field leakage issues. Therefore, this motivates the use of electromagnetic bandgap (EBG) structures to surround the periphery of the conventional waveguide to mitigate the risk or alleviate the adverse effects of field leakage. This is the essential idea of gap waveguide technology.

**Figure 3:**
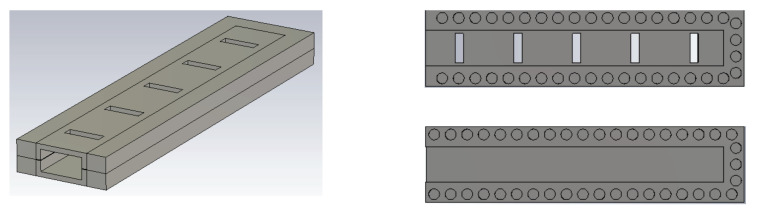
Designed slot array in groove gap waveguide implemented with holes.

**Figure 4:**
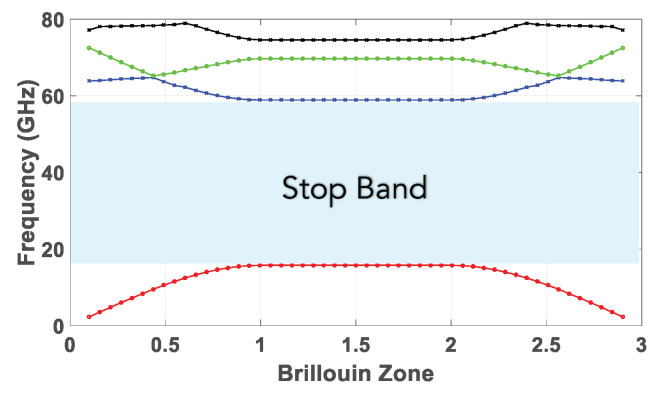
Dispersion diagram of the glide symmetric unit cell.

A perspective view of the groove gap waveguide with an array of five transverse slots cut out from its broad wall is depicted in Figure 3. Its interior layout showing the peripheral EBG structure as well as the lower and upper pieces are presented in Figure 3. As it can be seen, holes instead of pins are used to implement the groove gap waveguide. The reason is that, as mentioned in the introduction, these holes are much simpler to manufacture [12,13,21]. These holes must be in a glide symmetric configuration to obtain a bandgap. The dimensions of the holes have been calculated to have a stop band covering the Ka band. Particularly, the holes have a diameter of 2.7 mm, a periodicity of 4 mm, a depth of 2.1 mm, and the gap is assumed to be of 0.1 mm (although in practice the gap corresponds to the gaps created in between the two assembled pieces due to imperfections in the manufacturing). With these dimensions, the size of the created stopband goes from 17 to 59 GHz. The corresponding dispersion diagram of the unit cell used for the waveguide is shown in Figure 4. The eigen-mode solver in CST with periodic boundary conditions was used to produce this graph. Although an infinite array of such holes is entailed in theory, just a single row of them is enough to create an effective EBG effect for straight sections of waveguides, as already demonstrated in previous works of the authors [12,14,21].

**Figure 5:**
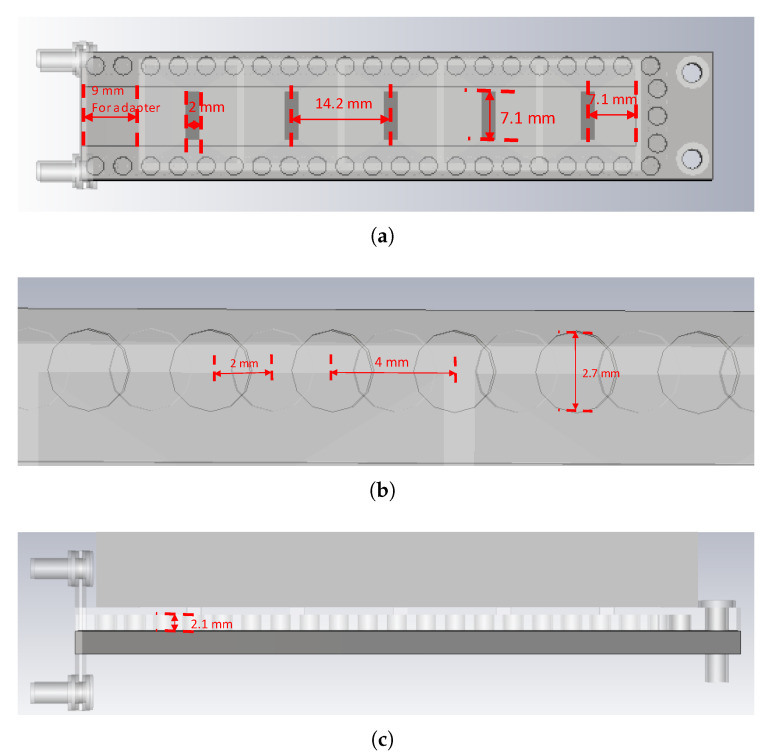
Geometrical details of the designed transverse waveguide slot array: (**a**) top view of the upper piece with slots; (**b**) zoomed-in view of part of the glide-symmetric hole-type electromagnetic bandgap (EBG); (**c**) side view.

The following section provides a more indepth description of the geometry. A schematic of the upper piece containing the slots is shown in Figure 5a. As depicted, the length and width of the slot are 7.1 and 2 mm, respectively. The periodicity is 14.2 mm and the distance from the terminal slot to the shorting metallic wall of the waveguide is half of this latter distance, as required. The 9 mm length of the adapter is also portrayed. A zoomed-in view of a portion of the waveguide wall lined with the glide-symmetric hole-type EBG structure is given in Figure 5b, displayed as two trains of overlapping circles, one for the lower piece and another for the upper one (one of them appears translucent). Both share a common period of 4 mm and they are displaced from each other by 2 mm. Finally, a lateral view of the structure is presented in Figure 5c in which the hole depth of 2.1 mm is conveyed.

**Figure 6:**
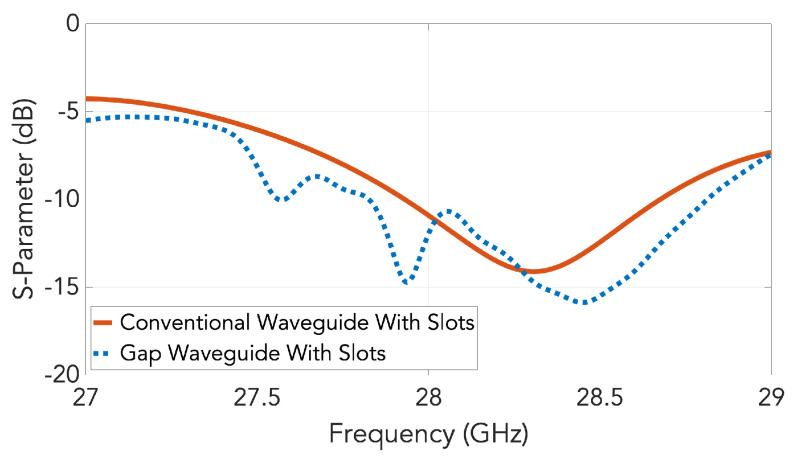
Simulated $S_{11}$ for the designed slot array in groove gap waveguide and in conventional waveguide.

Figure 6 shows the simulated $S_{11}$ of a conventional waveguide slot array using the simulation software *CST Microwave Studio*, demonstrating a designed operating band of 27.92–28.32 GHz if we assume that −10 dB of matching is needed. The result is seen to agree well with Figure 2 obtained from the TL model. In the same graph, the $S_{11}$ for the gap waveguide version is also included. A good agreement is observed. The non-smooth behavior in the gap waveguide version is due to numerical issues with the resonant periodic holey structure.

**Figure 7:**
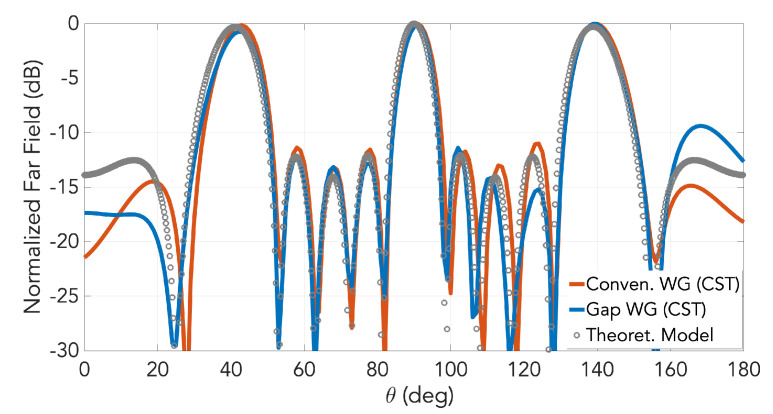
Radiation pattern in E-plane at 28 GHz for the designed slot array in groove gap waveguide and in conventional rectangular waveguide. Corresponding result of the theoretical model by antenna theory is included as circle markers.

The simulated far-field radiation patterns for both the waveguide slot array and the gap waveguide version are presented in Figure 7 for the frequency of 28 GHz. Only the pattern in E-plane is represented as it is here where we have the array behavior. Grating lobes are present in this plane for both cases because the inter-slot distance must be exactly one guided wavelength and that is longer than the wavelength in free space, as said earlier. Almost no differences are observed between the two cases. The corresponding radiation pattern computed using a mathematical field model based on aperture array antenna theory [22] (not presented here) is included in the figure as circle markers. As observed, very good agreement between this model and CST simulations is achieved.

## 4. Design of the Low Profile Horns

**Figure 8:**
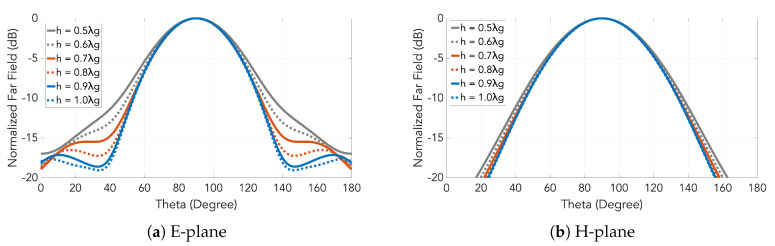
Radiation pattern of the designed horn as a function of the height *h*.

The next step is the design of the horns that are directly fed by the slotted waveguide presented in the previous section. The dimension of the horn in E-plane is defined by the the inter-slot spacing i.e., one guided wavelength. A good directivity is one of the targets in this design, but at the same time we want to keep the antenna as low profile as possible. Different heights for the horns were analyzed to study the effect of this parameter on the directivity, seen in Figure 8. Finally, a height of 0.5 $\lambda_{g}$ (7.1 mm) was selected as higher horns were not providing an increase in the directivity.

Considering the guided wavelength and the operation frequency, the grating lobes will appear at 49 degrees from the broadside direction. The radiation pattern of the horn must attenuate them at least 10 dB in the E-plane. All the heights fulfill this condition as seen in Figure 8.

**Figure 9:**
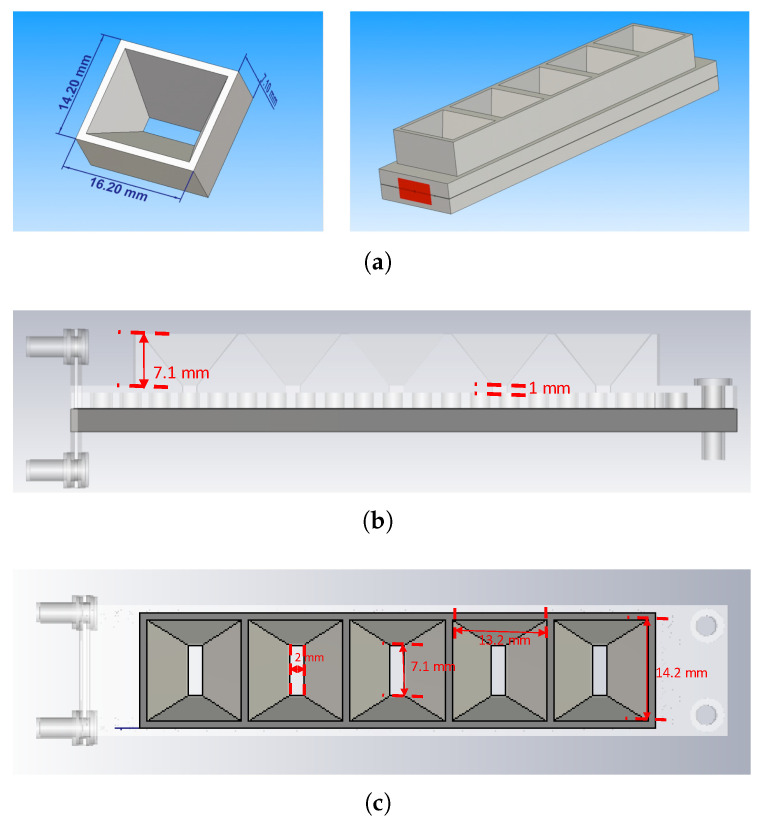
Designed horn and array of horns fed by the waveguide: (**a**) perspective views; (**b**) lateral view; (**c**) top view.

Schematics of the designed horn and the array composed of it are presented in Figure 9. In perspective views, Figure 9a shows the final dimensions of each horn and a representation of the linear horn array. The side view displaying the heights of the horn and waveguide wall is seen in Figure 9b, being 7.1 and 1 mm, respectively. Lastly, the lengths and breadths of the apertures of both the feeding slot and the horn are conveyed in Figure 9c as 7.1 by 2 mm for the slot and 14.2 by 13.2 mm for the horn.

**Figure 10:**
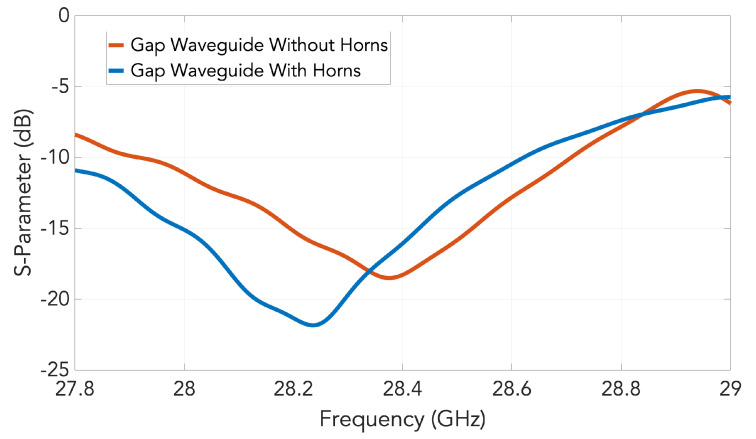
Simulated $S_{11}$ for the designed slot array in groove gap waveguide after adding the horns and comparison with the case without them.

The CST-simulated S-parameters of the gap waveguide slot array with and without the horns are shown in Figure 10. There is a slight change in the slot input impedance that actually improves the matching with the addition of the horns.

**Figure 11:**
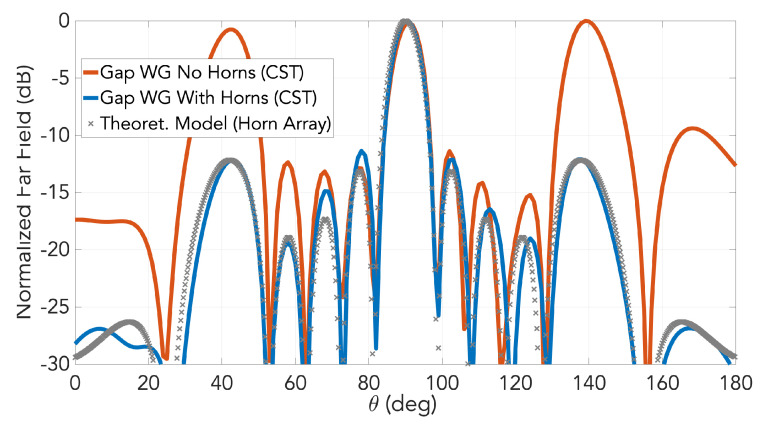
Comparison of the array radiation pattern in E-plane at 28 GHz between the cases with and without horns. Corresponding pattern computed from the theoretical model from [22] by horn array theory is included as cross markers.

The simulated far-field patterns in the E-plane of the slot array with horns is presented in Figure 11 for the frequency of 28 GHz. As observed, the grating lobes in E-plane have been successfully suppressed by the horns. For comparison, in the same figure the simulated radiation pattern for the slot array at the same frequency is shown in the same figure. The broadside lobe is almost identical for both designs. Included in the graph is the corresponding pattern computed by another mathematical field model (not presented here) based on horn array theory [22], conveyed by cross markers. Once again, excellent agreement with simulations was achieved by this model.

**Figure 12:**
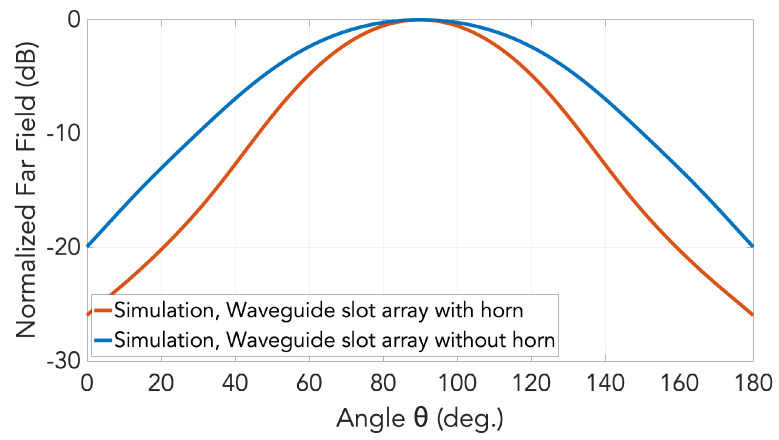
Comparison of the array radiation pattern in H-plane at 28 GHz between the cases with and without horns.

In addition to the E-plane patterns of Figure 11, those in H-planes for the cases with and without horns under the gap waveguide topology are also simulated and presented in Figure 12.

**Figure 13:**
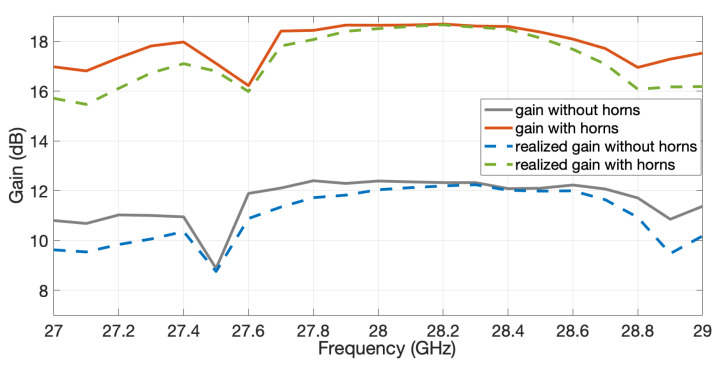
Comparison of the gain of the slot array and the horn array.

The comparison of the simulated gain and realized gain of the two arrays (slots and horns) as a function of the frequency is presented in Figure 13. At the central frequency, the gain reaches 18.6 dB when the horns are used in comparison with the 12.4 dB obtained with the slots.

## 5. Experimental Results

To manufacture the prototypes, some previous modifications are needed. A flange to attach the standard waveguide transition was added at one of the sides of the waveguide with the dimensions of the standard WR34 waveguide. Also, the waveguide was lengthened to allow for easy screwing. Additional screws were also added at the end of the structure to assemble the two pieces.

**Figure 14:**
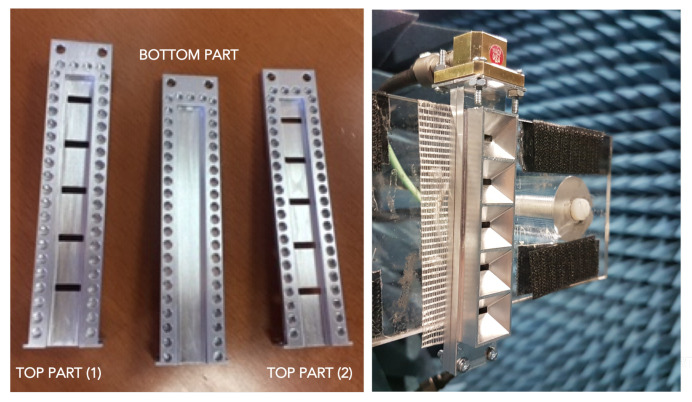
Manufactured prototypes.

Two prototypes were manufactured, one of them corresponding to the slot array and the second one including the horns. The two prototypes are shown in Figure 14. In the picture, the holey-EBG structure used to implement the gap waveguide concept can be seen in the inner part of the waveguide. Additionally it is also possible to observe the low profile characteristics of the horns.

**Figure 15:**
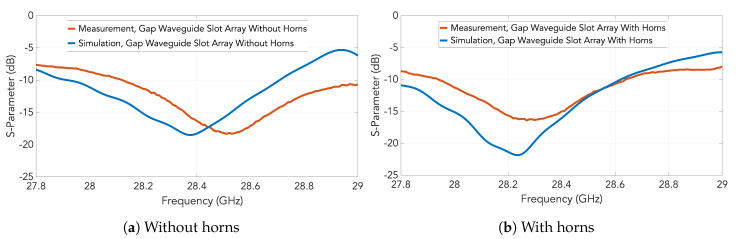
Measured $S_{11}$ for the designed slot array in groove gap waveguide shown by red traces: (**a**) with slots only without horns; (**b**) with slot-fed horns. In both plots, simulation results of Figure 10 are included as blue traces for comparison.

The measured $S_{11}$ parameters for the two prototypes are represented by red traces in Figure 15. Particularly, the case with the slots only without the horns is represented in Figure 15a whilst the case with the slot-fed horn array is included in Figure 15b. For comparison, the simulation results previously shown in Figure 10 are included as blue traces in the graphs. The low level in the whole band is probably caused by a bad contact in the measurement setup between the waveguide transition of the VNA and the flange of the antenna.

**Figure 16:**
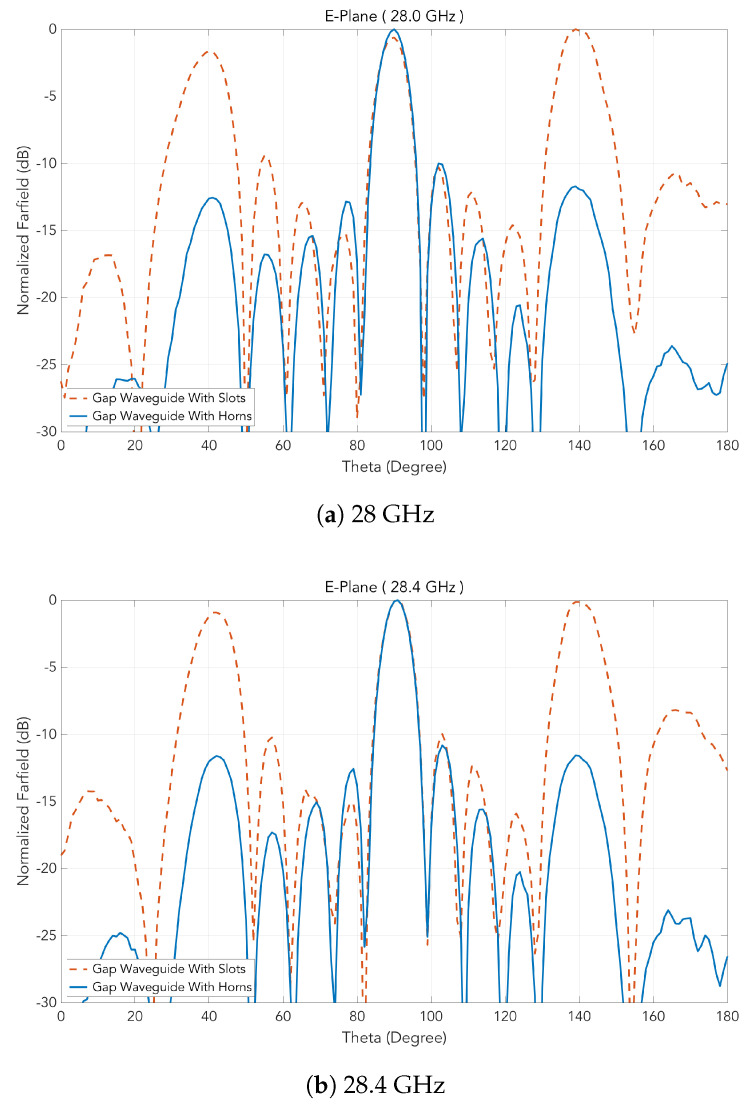

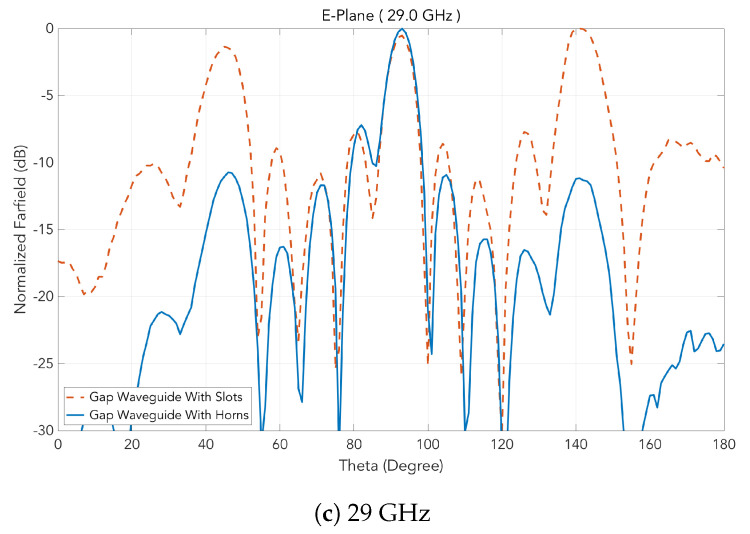
Measured H-plane at different frequencies for the two prototypes.

**Figure 17:**
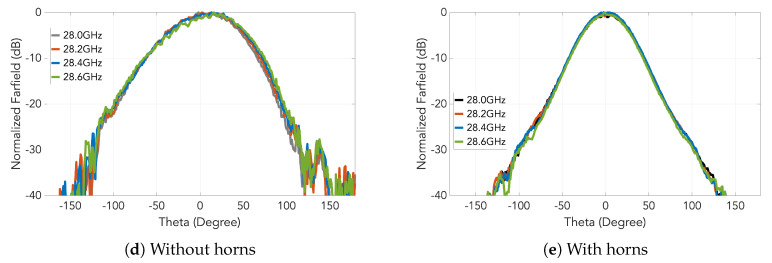
Measured E-plane at different frequencies for the two prototypes.

For both prototypes, the measured radiation patterns in E and H-planes are presented in Figure 16 and Figure 17, respectively, each of which at several frequencies. The suppression of the grating lobes is clear and a good agreement with the simulations can be observed.

**Figure 18:**
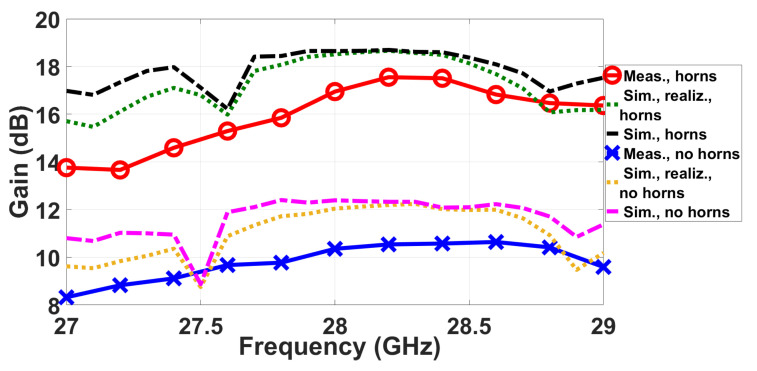
Measured gain for the two prototypes given as solid traces with markers. Broken traces (dashed or dotted) are corresponding simulated ones of Figure 13.

The measured realized gain for the two manufactured antennas as a function of the frequency is represented in Figure 18 by solid traces with markers; cross ones for the case without horns and circle markers for the case with horns. Plotted alongside for comparison are the corresponding simulated gains of Figure 13 shown as broken lines (dashed or dotted). The maximum gain is 17.55 dB for the case of the horns, i.e., 1 dB less than in simulations. This difference probably comes from a non-perfect contact between the transition used in the VNA and the flange of the antenna. This difference is even larger for the case of the slot array.

Finally, the attenuation of the grating lobes in the entire frequency range is more than 11 dB for both simulations and measurements.

**Figure 19:**
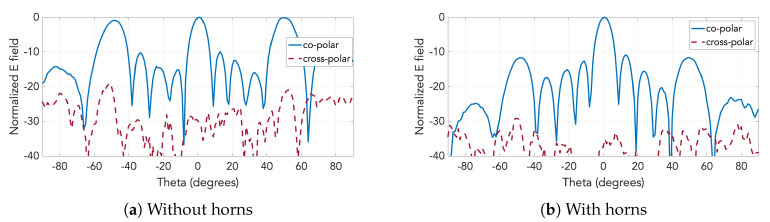
Measured co and cross polarization components in E-plane at 28.4 GHz for the two prototypes.

The cross-polarization component has also been also measured for the two antennas. Figure 19 contains the E-plane radiation patterns of the two prototypes including the cross-polarization component. While the level of cross polarization for the case with just the slots is already low, as Figure 19a shows, it gets even lower by the implementation of the horns, falling below −30 dB throughout the entire angular range, as seen in Figure 19b.

### Discussion

The contribution of this work focuses on the combination of low profile horns as in [9,23] with a feed system based on the groove version of gap waveguide technology. The selection of groove among the different versions has the advantage of providing the lowest losses as demonstrated in [8] and used in recent examples [24,25]. These losses are close to the ones provided by a standard rectangular waveguide. The advantage of the use of gap waveguide technology comes from the manufacturing, which can be made in two pieces that do not require a good electrical contact, just simply screwing when assembled together. This is a huge advantage when compared to standard waveguide technology where any imperfection or bad electrical contact can produce significant leakage. In this work, for the first time, the horns are fed by the groove gap waveguide version. In addition, the groove gap waveguide is implemented by a holey-EBG structure, which strongly reduces the manufacturing cost compared to the use of the typical pin-type EBG version.

From another perspective, the work can also be considered as a contribution to techniques for decreasing grating lobes in transverse slotted waveguide arrays. This is compared to former methods, such as solutions based on baffles comprising parallel plates were proposed in [2] as well as the use of pairs of slots in [26]. Another simple and straightforward approach is to reduce the guided wavelength by partial filling of the waveguide with a dielectric [27]. Also, in [28], unequal lengths of non-resonant transverse slots contained within a unit cell of period less than the free-space wavelength were used for phase compensation. The same concept of reflection cancellation to mitigate the grating lobes was used in [29,30], by adding in these cases parasitic dipole layers over the slot array. The use of low profile horns is the technique proposed here.

Table 1 contains a summary of the comparison of the proposed work with related works. Regarding the column for suppression of undesired lobes, some papers have no basis for comparison because their element spacings (array periodicities) are already small enough to prohibit the appearance of grating lobes at their frequencies of operation. It would be neither fair nor logical to compare our suppressed grating lobe level with even the highest sidelobe levels of those papers since the latter are still naturally going to be lower than grating lobes.

As can be seen, the performance levels of our design actually surpass those of numerous other papers in several aspects, such as fractional bandwidth, undesired lobe suppression, antenna efficiency, as well as the exemption from the need for dielectrics, which is vital at mm-wave and THz regimes where dielectric losses get intolerably severe.

## 6. Conclusions

An array of low profile horns fed by transverse slots etched on a groove gap waveguide has been presented. The design of the feeding groove gap waveguide was made using glide symmetric holes instead of pins to further simplify the manufacturing and decrease the cost. The designed horns are low profile and the whole prototype is made with simple milling of aluminum. As a consequence, the antenna has low losses.

The prototype was manufactured as two pieces, which were then assembled together later on by simple screwing using only four screws. The achieved gain is close to 18 dB for a linear array of only five elements. The design can be easily extended to a planar array version and has no limitations in the number of elements. In this sense it is much more flexible than standard array designs based on corporate feed networks that involve two-way power splits and with the number of power divisions being 2 raised to the power of n, where n is an integer.

The designed prototype for its flexibility, simplicity of manufacturing, and low losses is a good candidate to be used in 5G networks. Moreover, as the design is fully metallic, it can be easily scaled to higher frequencies without problems with its performances.

## Figures and Tables

**Table 1:** Comparison of performances with related works.

REF.	Fractional Bandwidth, Center Frequency	Undesired Lobe Suppression (dB)	Antenna Efficiency (dB)	Needs Dielectric
[31]	2.45%, 9.89 GHz	9	−3.15	Yes
[17]	3.4%, no info	8.1	−2.08	No
[32]	2.5%, 12 GHz	N.A.(no basis)	−3.07	No
[33]	1%, 9.5 GHz	N.A.(no basis)	−3.73	No
[34]	3.85%, 35.1 GHz	12	−2.433	Yes
[35]	1.3%, 76.5 GHz	13	no info	No
[36]	No info	N.A.(no basis)	−4.08	No
Ours	3%, 28.175 GHz	12	−2.46	No
